# Chinese herbal medicine for small cell lung cancer patients: A protocol for a systematic review and meta-analysis

**DOI:** 10.1097/MD.0000000000023746

**Published:** 2020-12-24

**Authors:** Xue Mi, Xiwen Zhang, Shulin He, Zhenhua Zhang, Runzhi Qi, Juling Jiang, Shuntai Chen, Honggang Zheng, Baojin Hua

**Affiliations:** a Department of Oncology, Guang’anmen Hospital, China Academy of Chinese Medical Sciences, Beijing; b Shaanxi University of Traditional Chinese Medicine, Xi’an; c Xichengqu Guangwai Hospital; d Graduate School, Beijing University of Chinese Medicine, Beijing, China.

**Keywords:** adverse events, Karnofsky, solid tumor, survival time, traditional chinese medicine

## Abstract

**Background:**

Small cell lung cancer (SCLC) is an aggressive disease. Chemotherapy is the standard treatment for SCLC, but the resistance and the adverse effects of Chemotherapy still remains a major problem. Although Chinese herbal medicine (traditional Chinese medicine) is wildly applied for patients with SCLC in China, the evidence of traditional Chinese medicine in the treatment for SCLC is limited.

**Method:**

We conducted a systematic search of PubMed, EMBASE, the Chinese National Knowledge Infrastructure, the VIP Information Database, and the Wanfang Database for relevant studies. Only randomized controlled trials were included. Two investigators independently reviewed the included studies and extracted relevant data. The effect estimate of interest was the relative risk or mean difference with 95% confidence intervals.

**Ethics and dissemination:**

Ethical approval is not required, as this study is based on the review of published research. This review will be published in a peer-reviewed journal and disseminated both electronically and in print.

**INPLASY registration number:**

INPLASY2020110055

## 1 Introduction

Lung cancer is the most common malignant tumor worldwide, with more than 2.09 million new cases in 2018.^[1]^ More than one-third of all newly diagnosed lung cancers were in China,^[2]^ which was the leading cause of cancer mortality for both men and women in the country.^[3]^ Small cell lung cancer (SCLC) accounts for 13% to 15% of all cases of lung cancer.^[4]^ SCLC is an aggressive disease. It is characterized by rapid growth and early metastatic spread to regional lymph nodes and distant sites. As for treatment, chemotherapy is the standard treatment for SCLC, because SCLC is very sensitive to chemotherapy.^[5]^ The majority of patients who usually exhibit sensitive responsiveness to chemotherapy relapse with relatively resistant disease very soon. Meantime, the side effects of chemotherapy seriously affect the treatment and quality of life of patients. According to the reports, about 80% of limited-stage small-cell lung cancer and almost all the extensive-stage small-cell lung cancer patients have a relapse or progression after treatment in 1 year, and approximately 95% of them eventually die from disease progression.^[6]^

In China, SCLC patients also use Chinese Herbal Medicine (TCM) during or after receiving chemotherapy.^[7]^ Some clinical trials have proved that TCM can be used to enhance the therapeutic effect of chemotherapy. TCM is also considered as an important supplementary therapy with beneficial effects for SCLC which can help reduce chemotherapy-related side effects and improve the quality of life.^[8]^ In recent years, a growing number of clinical studies that showed the efficacy and safety of TCM for SCLC were conducted^[9]^ (HY, 2001 #2). However, conclusions of these conducted trials based on relatively small sample size were sometimes conflicting. Therefore, we conducted this systematic review to evaluate the efficacy and safety of TCM adjuvant to chemotherapy for patients with SCLC.

## 2 Method

### 2.1 Study registration

**Figure 1:**
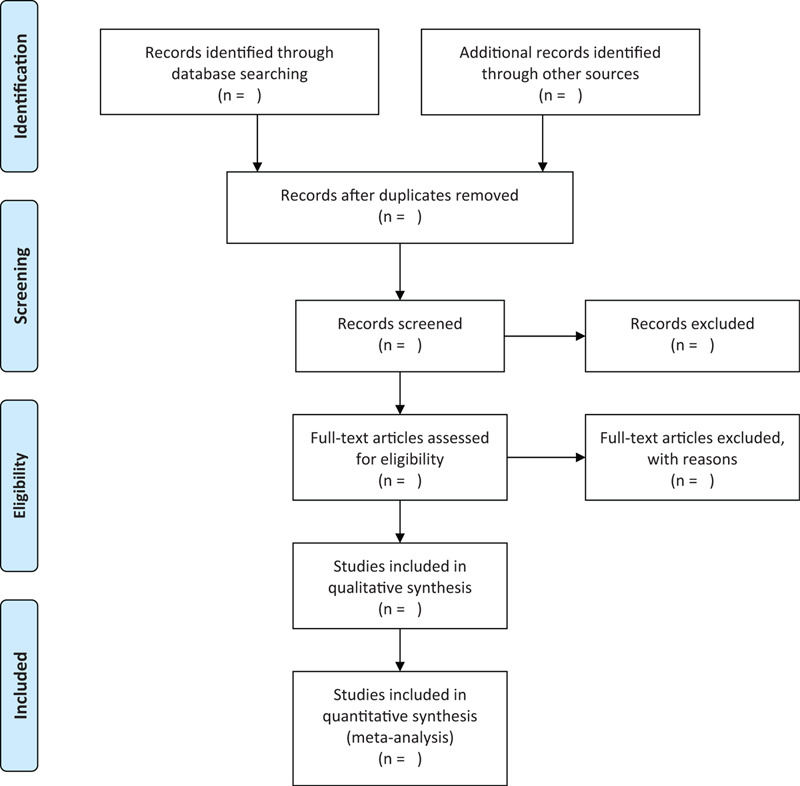
Flow diagram of studies search and selection.

This study will follow the guidelines outlined in the Preferred Reporting Items for Systematic Reviews and Meta-Analysis (PRISMA) statement for meta-analyses of healthcare interventions^[10]^; additionally, the protocol adheres to the PRISMA Protocols (PRISMA-P). The selection process will be summarized according to PRISMA flow diagram (Fig. 1).

The protocol for this systematic review has been registered on INPLASY under the number INPLASY2020110055

### 2.2 Types of studies

Randomized controlled trials regarding efficacy and safety of TCM in the treatment of SCLC will be included without restriction language.

### 2.3 Types of participants

Adult patients diagnosed with SCLC regardless of age, gender, and chemotherapy regimen. The diagnosis must have been confirmed by pathological or cytology diagnosis.

### 2.4 Types of interventions

According to the Pharmacopoeia of the People's Republic of China edited by the China Food and Drug Administration in 2015, TCM was defined as herbal agents and materials that originated from botanical herbal products, mineral and animal sources. The formulations of TCM included decoction, tablet, pill, powder, granule, capsule, oral liquid, and injection. Usually, a TCM formula is composed of 2 or more herbs to achieve synergistic effect for certain conditions, which is prescribed based on the traditional Chinese medicine pattern diagnosis and treatment thresholds by experienced physicians.

Participants in the TCM group should be treated by TCM and conventional chemotherapy. Participants in the control group should be treated by conventional chemotherapy alone or conventional chemotherapy plus placebo. No restrictions regarding number of herbs, formulations of TCM, or treatment duration was pre-established.

The specified exclusion criteria included:

(1) case reports, case series, reviews, editorials, commentaries, and animal studies;
(2) non-SCLC patients;
(3) other TCM complementary and alternative therapies, including acupuncture, moxibustion, cupping, massage, qigong, Tai Chi, and music therapy, were contained in either TCM or control group;
(4) duplication reporting the same results.

### 2.5 Types of outcome measures

The main outcome measure is therapeutic effect according to standard for therapeutic effect evaluation of solid tumor by response evaluation criteria in solid tumors.^[11]^ Second outcome measures are survival time, quality of life evaluated with Karnofsky score, and adverse events.

## 3 Search methods for the identification of studies

The Cochrane Library, MEDLINE, Embase, Chinese BioMedical Database (CBM), China National Knowledge Infrastructure, Chinese VIP Information (VIP), Wangfang Database will be searched regardless of publication date or language.

## 4 Data collection and analysis

### 4.1 Selection of studies and data extraction

Two reviewers independently selected studies based on the above inclusion and exclusion criteria. The following details from each trial were independently extracted by 2 reviewers:

(1) general information: the title of the study, the first author's name, and publication year;
(2) the characteristics of the participants: age, sample size, and baseline information;
(3) the characteristics of the included trials: methodological design, interventions in the treatment and control groups, compositions and dosage of TCM and chemotherapy, and treatment duration;
(4) both primary and secondary outcome measures; and
(5) adverse effects.

The original correspondence authors were contacted via email, fax, and telephone for further information if missing data was identified. If no response was got, we either measured data from the graphs using digital ruler software or excluded it accordingly. Disagreements on data extraction were resolved through consultation with the third party.

### 4.2 Assessment of risk of bias in included studies

Two authors will independently assess the methodological quality of included trials. The methodological quality of the included randomized controlled trials will be assessed according to the guidance of the Cochrane Handbook for Systematic Review of Interventions, Version 5.1.0,^[12]^ which includes the following 7 criteria: random sequence generation (selection bias), allocation concealment (selection bias), blinding of participants and personnel (performance bias), blinding of outcome assessments (detection bias), incomplete outcome data (attrition bias), selective outcome reporting (reporting bias), and other sources of bias. Consensus will be reached by discussion with a third author in case of discrepancies. If necessary, we will contact the authors for missing data, methods of blinding, and randomization.

### 4.3 Measures of treatment effect

We will apply relative risk to represent the enumeration data; measurement data will be represented by mean difference and 95% confidence interval.

### 4.4 Dealing with missing data

Corresponding authors will be connected by E-mail for detailed data if their studies’ information is not available. If no additional message is received, we will conduct data synthesis using available data.

### 4.5 Assessment of quality in included studies

The quality of each selected studies will be evaluated using the Grading of Recommendations Assessment, Development, and Evaluation^[13]^ approach by 3 investigators.

### 4.6 Assessment of heterogeneity

Random models will be applied to conduct the meta-analysis. we will use Chi-squared and *I*^2^ tests to evaluate the heterogeneity of all studies included. *I*^2^ values >50 means high heterogeneity among studies included. If there is a high heterogeneity, we will conduct subgroup analyses to explore the possible causes

### 4.7 Assessment of reporting bias

If there are more than 10 included trials in this review, funnel plot will be used to discuss the reporting biases or small-study effects according to Egger methods.

### 4.8 Data synthesis

We will use RevMan 5.3 software (The Cochrane Collaboration, Oxford, England) to calculate for data synthesis. If there no obvious statistical heterogeneity among the trials included, we will apply fixed effects model to perform in the analysis. However, the random-effects model will be used, when apparent clinical heterogeneity among the trials included. Meanwhile, subgroup or sensitivity analysis will be conducted. α = 0.05 will be deemed statistically significant.

### 4.9 Subgroup analysis

Subgroup analysis will be conducted according to sex, smoking status, locations, histologic diagnosis, TNM stage, duration of TCM therapies, timing of TCM therapies, chemotherapy regimens.

### 4.10 Sensitivity analysis

Sensitivity analysis will be conducted to explore the quality of studies of the document following sample size, the outcome of missing data, and methodological quality.

### 4.11 Ethics and dissemination

Ethical approval is not required because individual patient information will be not used. The authors will disseminate This systematic review through conference presentations and peer-review publications

## 5 Discussion

Although, some clinical trials have proved that TCM can be used to enhance the therapeutic effect of chemotherapy. TCM is also considered an important supplementary therapy with beneficial effects for SCLC which can help reduce chemotherapy-related side effects and improve the quality of life. In recent years, a growing number of clinical studies that showed the efficacy and safety of TCM for SCLC were conducted. However, conclusions of these conducted trials based on relatively small sample size were sometimes conflicting. Therefore, this protocol for a systematic review has to be displayed. We hope that our work will help clinicians with more convincing evidence to deal with patients with SCLC.

## Author contributions

**Conceptualization:** Mi Xue, Shuntai Chen, Honggang Zheng, Baojin Hua.

**Data curation:** Mi Xue, Xiwen Zhang, Shulin He, Runzhi Qi, Juling Jiang.

**Formal analysis:** Mi Xue.

**Methodology:** Mi Xue, Xiwen Zhang.

**Resources:** Shulin He, Zhenhua Zhang, Honggang Zheng.

**Software:** Mi Xue, Zhenhua Zhang, Runzhi Qi, Juling Jiang.

**Writing – original draft:** Mi Xue, Shuntai Chen.

**Writing – review & editing:** Mi Xue, Shuntai Chen, Baojin Hua.
